# Protective effect of titanium tetrafluoride and silver diamine fluoride on radiation-induced dentin caries in vitro

**DOI:** 10.1038/s41598-021-85748-8

**Published:** 2021-03-16

**Authors:** Beatriz Martines de Souza, Mayara Souza Silva, Aline Silva Braga, Patrícia Sanches Kerges Bueno, Paulo Sergio da Silva Santos, Marília Afonso Rabelo Buzalaf, Ana Carolina Magalhães

**Affiliations:** 1 Department of Biological Sciences, Bauru School of Dentistry, University of São Paulo, Alameda Octávio Pinheiro Brisolla 9-75, Bauru, São Paulo 17012-191 Brazil; 2 Department of Surgery, Stomatology, Pathology and Radiology, Bauru School of Dentistry, University of São Paulo, Alameda Octávio Pinheiro Brisolla 9-75, Bauru, São Paulo 17012-191 Brazil

**Keywords:** Microbiology, Cancer, Dental diseases

## Abstract

This in vitro study evaluated the protective effect of titanium tetrafluoride (TiF_4_) varnish and silver diamine fluoride (SDF) solution on the radiation-induced dentin caries. Bovine root dentin samples were irradiated (70 Gy) and treated as follows: (6 h): 4% TiF_4_ varnish; 5.42% NaF varnish; 30% SDF solution; placebo varnish; or untreated (negative control). Microcosm biofilm was produced from human dental biofilm (from patients with head-neck cancer) mixed with McBain saliva for the first 8 h. After 16 h and from day 2 to day 5, McBain saliva (0.2% sucrose) was replaced daily (37 °C, 5% CO_2_) (biological triplicate). Demineralization was quantified by transverse microradiography (TMR), while biofilm was analyzed by using viability, colony-forming units (CFU) counting and lactic acid production assays. The data were statistically analyzed by ANOVA (*p* < 0.05). TiF_4_ and SDF were able to reduce mineral loss compared to placebo and the negative control. TiF_4_ and SDF significantly reduced the biofilm viability compared to negative control. TiF_4_ significantly reduced the CFU count of total microorganism, while only SDF affected total streptococci and mutans streptococci counts. The varnishes induced a reduction in lactic acid production compared to the negative control. TiF_4_ and SDF may be good alternatives to control the development of radiation-induced dentin caries.

## Introduction

Head and neck cancer (HNC) represents the sixth most common type of cancer diagnosed worldwide. Unfortunately, more than 66% of HNC cases are diagnosed in advanced stages (III or IV)^1^. The radiotherapy, associated or not with other therapies, is the main treatment for malignant HNC lesions^2^, based on the use of high doses of X-rays to destroy tumor cells. However, normal cells are also affected by head and neck radiotherapy, which can cause salivary gland dysfunction and, consequently, hyposalivation that dramatically increases the risk for dental caries. Furthermore, radiotherapy also causes some damage to the dental hard tissue, increasing its susceptibility to demineralization^3,4^.

Root caries lesions (RCLs) are often diagnosed with advanced age, due to hyposalivation and to the root exposure caused by gingival recession that results from aggressive toothbrushing or chronic periodontitis^5^. Global annual RCL incidence is reported to vary from 10.1 to 40.6% for healthy people^6^, while for patients with head and neck radiotherapy is about 16% after the first year, reaching up to 74% after 7 years of treatment^7,8^. Therefore, interventions to prevent this type of dental disease are needed.

Radiation-induced dental caries is a complex and multifactorial disease, which differs from conventional dental caries due to its sudden progression, rapidly compromising dentin, and reaching surfaces that are not usually affected by carious lesions, such as tips of cusps and smooth surfaces^9^. Depending on the radiotherapy site, teeth can be significantly affected by the maximum dosage (around 99% in case of tongue tumors, for example)^10^. Both enamel and dentin are already softened by 10 Gy^11–13^, a dose much lower than those applied in case of HNC. A recent study has shown that radiotherapy decreases odontoblastic cell metabolism, caused by decreased vascularization, as well as induces degradation of collagen fibers^9^.

Fluorides have been used to prevent RCLs. Annual application of sodium fluoride (5%) varnish or 38% silver diamine fluoride (SDF) solution are able to reduce the emergence of new RCLs by 64 and 71%, respectively^14^. The application of fluoride products on the teeth has been suggested throughout the period of radiotherapy^15–17^, however, there are few studies on this subject. Wu et al.^18^ showed in vitro that dentin (exposed to 68.25 Gy of radiation) treated daily with 5% NaF varnish, effectively showed a restored surface and increased microhardness compared to untreated irradiated dentin.

On the other hand, previous studies have shown a promising effect of titanium tetrafluoride (TiF_4_) varnish to control enamel de-remineralization compared to NaF varnish^19,20^. The application of TiF_4_ induces deposition of an acid-resistant layer containing titanium oxide and hydrated titanium phosphate, able to provide mechanical protection, and higher fluoride uptake compared to NaF, increasing the acid resistance of dental hard tissue^21^.

A recent study also showed a promising effect of TiF_4_ varnish on the prevention of dentin carious lesions formation compared to NaF varnish, under microcosm biofilm model produced on previous sound dentin^22^. We do not know if TiF_4_ varnish would have the same protective effect if applied on previous irradiated dentin, as well as under conditions simulating patients with HNC. The same is assumed for SDF.

Due to the need for finding alternatives to minimize the development of radiation-induced dentin caries, the aim of this work was to evaluate: (1) the protective effect of TiF_4_ varnish compared to NaF varnish and SDF solution on irradiated root dentin with respect to demineralization; and (2) the antimicrobial action of TiF_4_ varnish compared to NaF varnish and SDF solution on microcosm biofilm produced from biofilm collected from patients subjected to head and neck radiation.

## Results

**Figure 1:**
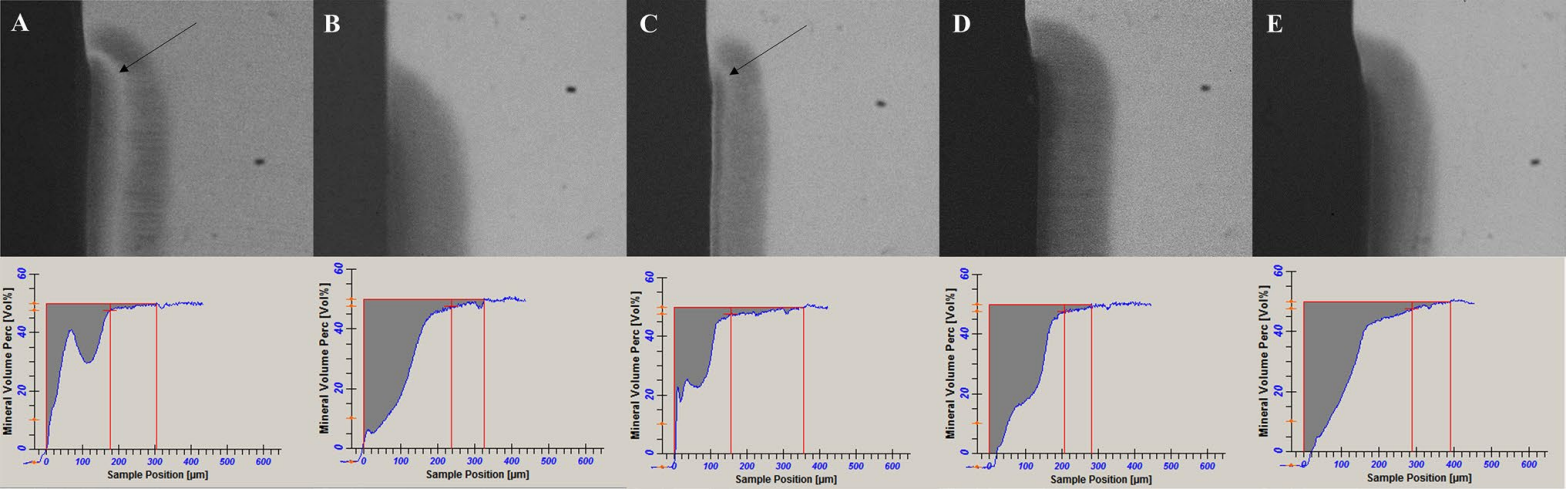
Representative TMR image and lesion profile of a dentin sample from each of the following groups: (**A**) TiF_4_ (**B**) NaF (**C**) SDF (**D**) Placebo (**E**) Negative control. *Arrows identify mineralized areas (radiopaque area) in the lesions belong to TiF_4_ varnish and SDF solution groups.

TiF_4_ varnish and SDF solution significantly reduced the integrated mineral loss compared to NaF varnish, placebo varnish and negative control (*p* < 0.0001). With respect to lesion depth, no protective effect of fluorides was found (Table 1). Figure 1 shows the TMR image and the lesion profile of a representative dentin sample per group.

**Table 1:** Mean and standard deviation of the integrated mineral loss (ΔZ, vol% μm) and lesion depth (LD, μm) presented by the demineralized dentin samples.

Treatments	ΔZ (vol%.μm)	LD (μm)
TiF_4_	3266 ± 1173^A^	180 ± 64^A^
NaF	5686 ± 802^B^	199 ± 25^A^
SDF	3396 ± 831^A^	200 ± 36^A^
Placebo	5692 ± 707^B^	201 ± 28^A^
Negative control	5366 ± 786^B^	203 ± 40^A^

Different letters in the same column indicate significant differences among the treatments. ANOVA and Tukey–Kramer test (ΔZ *p* < 0.0001; LD *p* = 0.3986).

**Figure 2:**
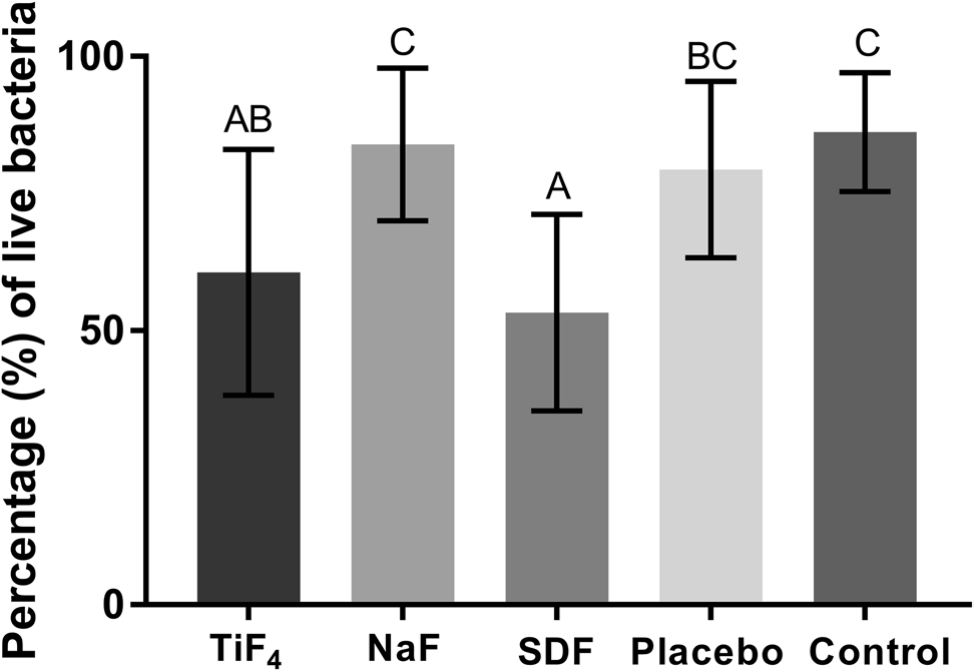
Mean and standard deviation of the percentage of live bacteria (%). Different letters show significant differences among the treatments. ANOVA/ Tukey–Kramer test (*p* < 0.0001).

TiF_4_ and SDF significantly reduced biofilm viability compared to negative control, while SDF further differed from placebo varnish (*p* < 0.0001). NaF had no effect on biofilm viability (Fig. 2). TiF_4_ and NaF varnishes-treated biofilms had reduced thickness (17.9 ± 3.1 and 18.7 ± 3.3 μm, respectively) compared to negative control (24.3 ± 4.3 μm), but not to placebo varnish (20.3 ± 3.6 μm) (*p* = 0.0032). SDF had no effect on biofilm thickness (20.0 ± 3.7 μm).

In agreement with viability assay, TiF_4_ was the only one able to reduce the CFU counts of total microorganisms (*p* = 0.003). No effect of fluoride treatments was seen on *Lactobacillus* sp. SDF significantly reduced the total streptococci (*p* < 0.0001) and mutans streptococci (*p* = 0.0001) CFU numbers compared to placebo varnish and negative control, while TiF_4_ and NaF did not (Table 2).

**Table 2:** Mean and standard deviation of the colony forming unit (CFU) counting (log_10_ CFU/mL) for total microorganisms, total streptococci, mutans streptococci and *Lactobacillus* sp*.*

Treatments	Total microorganisms	Total streptococci	Mutans streptococci	*Lactobacillus* sp.
TiF_4_	6.86 ± 0.21^A^	6.27 ± 0.27^AB^	6.52 ± 0.19^AB^	6.51 ± 0.40^A^
NaF	7.02 ± 0.31^AB^	6.69 ± 0.41^BC^	6.80 ± 0.30^B^	6.63 ± 0.32^A^
SDF	7.01 ± 0.20^AB^	6.07 ± 0.53^A^	6.29 ± 0.38^A^	6.73 ± 0.28^A^
Placebo	7.21 ± 0.23^B^	7.00 ± 0.32^C^	6.77 ± 0.25^B^	6.82 ± 0.44^A^
Negative control	7.16 ± 0.15^B^	6.59 ± 0.29^BC^	6.69 ± 0.22^B^	6.75 ± 0.27^A^

Different letters in the same column indicate significant differences among the treatments. ANOVA and Tukey–Kramer test (total microorganisms *p* = 0.003; total streptococci *p* < 0.0001; mutans streptococci *p* = 0.0001 and; *Lactobacillus* sp*.* *p* = 0.23).

**Figure 3:**
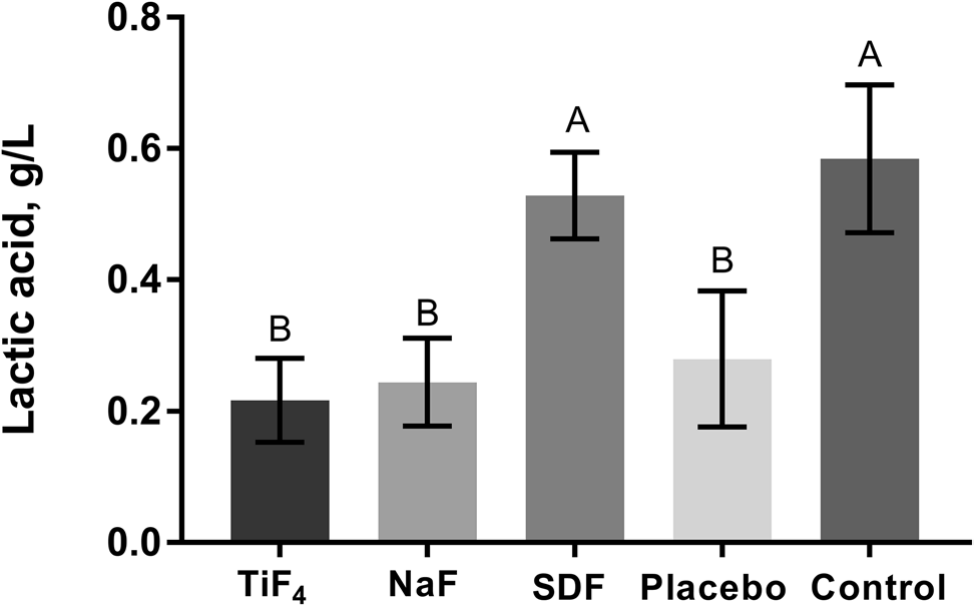
Mean and standard deviation of the amount of produced lactic acid (g/L) by the biofilm. Different letters show significant differences among the treatments. ANOVA/ Tukey–Kramer test (*p* < 0.0001).

All varnishes (TiF_4_, NaF and placebo) significantly reduced lactic acid biofilm production compared to negative control and SDF (*p* < 0.0001), which in turn did not differ from each other (Fig. 3).

## Discussion

Our recent study has shown irrelevant antimicrobial effect of TiF_4_ varnish, but a significant ability to reduce carious lesions development in sound dentin under microcosm biofilm model^22^. One other recent study evaluated the effect of fluorides on irradiated dentin, but not under cariogenic conditions^18^.

Considering the high prevalence of HNC and the side-effects of radiotherapy, efforts to find a good alternative to avoid the development of radiation-dentin caries lesions are extremely relevant. Accordingly, our study confirmed the anti-caries effect of TiF_4_ varnish compared to NaF varnish, as shown by Dos Santos et al.^22^, which was comparable to those found for SDF. An annual application of SDF is able to prevent 71% root caries lesions development^14^. However, tooth discoloration induced by SDF application is of major concern, while no significant staining potential by TiF_4_ varnish was found^23^.

Interestingly, carious lesions created under conditions simulating irradiated patients were 1.5–2-folder greater compared to those induced on sound dentin under a similar microcosm biofilm^22^ and, despite it, TiF_4_ varnish still had a protective effect. This result is also supported by an in vivo study^24^, which further showed penetration of F and Ti into the dentin caries lesion. According to Tveit et al.^25^, a titanium-rich layer remained on the dentin surface even after 3 weeks of TiF_4_ application in vivo.

Microcosm biofilm induced using human biofilm from irradiated patients also presented a bacterial load 1.2–1.4 times higher compared to microcosm biofilm growth under health conditions on dentin samples^22^. Signori et al.^26^ have discussed that the bacteria source (saliva vs. biofilm, caries vs. free-caries patient) has no influence on the potential of microcosm biofilm to induce demineralization under sucrose exposure. However, in the present study, the bacterial load may have had a significant influence on radiation-induced dentin caries. We thus speculate that a high bacterial load could have impaired the anti-caries effect of NaF, found in our previous work^22^, but not demonstrated here.

Therefore, we are planning to conduct future studies analyzing the biofilm behavior and the degree of dentin demineralization, comparing the sources of bacteria (biofilm from irradiated vs. non irradiated patients) and the quality of dentin substrate (irradiated vs. non irradiated), to better address the effect of both factors on the caries development.

We did not apply methods to demonstrate morphological dentin alterations induced by radiation and its relation to the caries lesions formation. We have support from previous studies that showed morphological dentin alterations (presence of cracks on surface and dentin tubules occlusion) after irradiation, by using scanning electron microscopy-SEM^18,27^. The damage of dentin surface induced by irradiation can have interfered on the CaF_2_ deposition produced by NaF varnish, and consequently on the F^−^ release from this reservoir, justifying its lack of antimicrobial and anti-caries effects in the present study either.

In contrast to the report of Dos Santos et al.^22^, the present study found that TiF_4_ was able to reduce total microorganism CFU, however, with no effect on the numbers of cariogenic species. A microbiome study of microcosm biofilm growth under health and radiation conditions are needed, since other species not studied here may be part of the microcosm biofilm, contributing to the sudden caries lesion development in irradiated dentin^28^ and justifying the antimicrobial effect of TiF_4_ varnish found on total microorganisms.

SDF, on the other hand, had an antimicrobial effect on total streptococci and mutans streptococci, in agreement with the literature^29,30^. Zhao et al.^30^ demonstrated that SDF hinders dentin collagen degradation. SDF also induces the formation of products such as CaF_2_, Ag_3_PO_4_ and NH_4_OH, which interact with dentin hydroxyapatite forming fluorapatite^31^, providing an acid-resistant structure. A clinical trial also confirms that SDF is more effective in stopping caries lesions in dentin when compared to NaF^32^. Therefore, the anti-caries effect of SDF may be due to its antimicrobial action and also to its chemical interaction with dentin surface. It is likely that the interaction with the tooth surface is more important than its antimicrobial effect, since SDF did not reduce lactate production, despite it had significantly decreased the cariogenic microorganism CFU counting.

In principle, we expected to see low lactate production by the biofilm after SDF application, since this fluoride significantly decreased mutans streptococci CFU counting^33^, which was not observed. We speculate that other aciduric bacteria could be presented in the biofilm, which in turn were not affected by SDF. Also, SDF did not reduce the biofilm thickness. The high amount of extracellular matrix, involved in the biofilm thickness, could have retained more acid in the biofilm, justifying our findings. This hypothesis needs to be confirmed in future studies.

NaF varnish has previously shown to reduce the production of lactic acid under microcosm biofilm^34^, mono-species biofilm^35^ and multispecies biofilm^36^. Both NaF and TiF_4_ significantly reduced biofilm thickness and lactic acid production, despite the fact that they were not different from placebo varnish, in disagreement with the findings of Dos Santos et al.^22^ This finding deserves further attention. Somehow component(s) of our varnish (except F) might have acted as glycolytic enzyme inhibitor(s), reducing the amount of lactate produced by the biofilm.

The caries protective effect of TiF_4_ seems to be more due to dentin surface modification than to the antimicrobial effect. Interestingly, the TMR profile of TiF_4_-treated dentin showed an intermediated highly mineralized layer (around 90 μm depth), which might contain F and Ti. For both TiF_4_ and SDF, their chemical interaction could have improved mineral gain especially in the intermediate layer (arrows in Fig. 1), but they were not able to impair bacterial acid penetration and, consequently, no reduction in lesion depth was seen.

In conclusion, both TiF_4_ varnish and SDF solution were similarly able to reduce the development of radiation-induced dentin caries in vitro. The result of the present study needs to be confirmed by randomized clinical trials in patients affected by HNC and treated with radiotherapy.

## Methods

### Tooth sample preparation and treatment groups

The bovine teeth were collected from cattle slaughtered in the food manufacturing industry (Frigol S.A, Lençóis Paulista-SP, Brazil). The study was approved by Ethics committee on animal research (CEUA, Number: 004/2018, Bauru School of Dentistry, University of São Paulo, Bauru, Brazil) following the guidelines of the CONCEA (National Council for Control of Animal Experimentation). No animals were harmed in order to conduct this study. One hundred and eighty bovine root dentin samples (4 mm × 4 mm) were prepared^22^. Dentin samples were submitted to X-rays from linear accelerator with an energy of 6 meV (Varian, Clinac 6EX, USA) in total dose of 70 Gy and, thereafter, sterilized using ethylene oxide for 4 h under a pressure of 0.5 ± 0.1 kgF/cm.

Before sterilization, the average surface roughness was measured by using a contact profilometer (Mahr Perthometer, Göttingen, Germany) and the software MarSurf XCR-20 (Mahr Perthometer, Göttingen, Germany) (5 readings for the calculation of the mean), for samples randomization into the groups (n = 36/group, n = 12 for each biofilm assay): 4% TiF_4_ varnish (pH 1.0, 2.45% F); 5.42% NaF varnish (pH 5.0, 2.45% F); 30% SDF solution (pH 8.5, 3.54% F); placebo varnish (pH 5.0) or untreated (negative control)^37^. The dentin roughness may influence biofilm formation^22^ and, therefore, it was applied to standardize the initial conditions of the groups (mean: 0.34 ± 0.03 μm). Before treatment, 2/3 of the dentin surface was protected using nail polish to allow to have 2 control areas (untreated and non-demineralized areas).

The F and placebo varnishes contained the same artificial resin as base and ethanol as solvent; SDF solution contained hydrofluoric acid, silver nitrate, ammonium hydroxide and deionized water. The treatments were applied using microbrush on the samples surfaces for 6 h. During this period, dentin samples were stored in remineralizing solution^37^. The treatments were then removed using cotton swab and acetone solution^22,37^ and the nail polish was reapplied at the same sites, before biofilm formation.

### Microcosm biofilm formation

A mixed solution containing thawed inoculum (compound from human biofilm from 2 donors who received a total radiation dose of 70 Gy in the head and neck region, mixed with 1% saline solution in the proportion 2 g: 1 mL, and diluted for freezing in 30% of glycerol^26^) and McBain saliva (proportion 1:50) was added to each well containing a treated dentin samples (24-well microplate, 1.5 mL/well), and incubated for 8 h (5% CO_2_ and 37 °C)^22,38^. Thereafter, the medium was removed and fresh McBain saliva containing 0.2% sucrose was added to the wells for further 16 h^22,37^. The medium was replaced daily for more 4 days and incubated under the same conditions described above^22,37^.

### Demineralization analysis: Transverse microradiography (TMR)

After 5 days of biofilm growth, dentin samples (except those from the lactic acid assay) were cleaned, transversally sectioned, polished and submitted to microradiograph exposure (20 kV and 20 mA, Softex, Tokyo, Japan) as previously described^22^. The developed plate was analyzed using a transmitted light microscope fitted with a 20× objective. Two images per sample were obtained using data acquisition (version 2012) and interpreted using calculation (version 2006) software from Inspektor Research System (Amsterdam, Netherlands). The mineral content was calculated based on the work of Dos Santos et al*.*^22^, assuming 50 vol% of mineral content for sound dentin and that the lesion depth ends when dentin contains around of 47.5% of mineral volume. The integrated mineral loss (ΔZ, vol% μm) and lesion depth (LD, μm) were calculated for the mean of the 2 images per sample.

### Biofilm viability analysis

Biofilm was stained using nucleic acid marker (2 µM SYTO 9 *green fluorescent nucleic acid stain*, Thermo Fisher Scientific, USA) (v = 10 μL/well) for 15 min in a dark environment^22^. Confocal laser scanning microscopy (Leica TCS SPE, Mannheim, Germany) and Leica Application Suite-Advanced Fluorescence software (LAS AF, Mannheim, Germany) were used to analyze the biofilm surface. Three images (275 μm^2^) were captured and analyzed using BioImage L 2.0 software. The percentage of live bacteria-% and the biofilm thickness-μm were obtained.

### Analysis of colony forming units (CFU)

Four different types of agar were used to the CFU counting: 1) brain heart infusion agar (BHI; Difco, Detroit, USA) for total microorganisms; (2) mitis salivarius agar (MSA; Neogen, Indaiatuba, Brazil) for total streptococci; (3) SB-20 M for mutans streptococci; and (4) rogosa (MRS agar; Kasvi, Curitiba, Brazil) for *Lactobacillus* sp.^22^. Bacterial suspensions were diluted (10^−4^) and spread on Petri dishes (25 μL/dish) and then, the dishes incubated under 5% CO_2_ and 37 °C for 48 h. The CFU numbers were counted by two examiners and converted to log_10_ CFU/mL.

### Analysis of lactic acid production

Dentin samples with 5-day microcosm biofilm were incubated in a buffered peptone water (BPW) (Synth, Diadema, Brazil) supplemented with 0.2% sucrose (v = 1 mL/sample) for 3 h, under 5% CO_2_ and 37 °C^22^. Lactate concentrations were evidenced via enzymatic method (Enzymatic assay for d- and l-Lactic acid—Ref. 8240; R-Biopharm, Darmstadt, Germany) following the manufacturer’s guidelines. The absorbance was measured at 340 nm using a microplate reader (Fluorstar Optima—BMG Labtech, Ortenberg, Germany) and the values converted to g/L.

### Statistical analysis

The biofilm assays (viability, UFC counting and lactic acid assay) were performed in triplicate with four data points for each replicate (n = 12). Dentin samples from all biofilm analysis, except from the lactic acid assay, were analyzed by TMR (n = 24). Data were statistically compared using GraphPad Prism software for Windows (GraphPad Software, San Diego, USA). The normal distribution and homogeneity were checked using Kolmogorov–Smirnov and Bartlett tests, respectively. All data were compared using Analysis of Variance (ANOVA) followed by Tukey–Kramer test. The level of significance was set at 5%.

### Ethics aspects and Saliva collection

The local ethical committee (CAAE: 97497318.00000.5417) of Bauru School of Dentistry-USP (Bauru-Brazil) approved this study. The study was conducted in accordance with the ethical standards of the Institutional and/or National Research Committee and with the 1964 Helsinki declaration and its later amendments or comparable ethical standards. Written informed consent was obtained from all individual participants included in the study. Biofilm was collected from two donors (1 male: 65 years old with 20 teeth and 1 female: 57 years old with 24 teeth) who received the total head and neck 3D radiotherapy (final dose: 70 Gy), 5 months previously to the study, and met the inclusion criteria: (1) low salivary flow (stimulated saliva flow < 1 mL/min and non-stimulated saliva flow < 0.3 mL/min), (2) without acute gingivitis, (3) not using antibiotics or (4) being submitted to professional fluoride application in the last 3 months neither. Biofilm was collected from the cervical area of all roots without active caries lesions, by using a periodontal curette. Biofilm collection and storage were performed as described by Signore et al.^26^.
